# Next-Generation Sequencing Reveals the Potential Role of RET Protooncogene in Metastasis Progression in Medullary Thyroid Cancer

**DOI:** 10.3390/cimb47070560

**Published:** 2025-07-18

**Authors:** Maurice Klein, Anna Julia Claudia Klein, Arnold M. Raem, Nicklas Garrelfs, Henrike J. Fischer, Frank Hölzle, Kai Wermker

**Affiliations:** 1 Department of Oral & Maxillofacial Surgery, School of Medicine, University Hospital RWTH Aachen, Pauwelsstrasse 30, 52074 Aachen, Germany; 2 Department of Otolaryngology and Head and Neck Surgery, School of Medicine, University Hospital RWTH Aachen, Pauwelsstrasse 30, 52074 Aachen, Germany; 3 Arrows Biomedical Deutschland GmbH, CeNTech Heisenbergstraße 11, 48149 Münster, Germany; 4 Department of Intensive and Intermediate Care, Pauwelsstrasse 30, 52074 Aachen, Germany; 5 Institute of Immunology, Medical Faculty, RWTH Aachen University, Pauwelsstr. 30, 52074 Aachen, Germany; 6 Department of Oral and Cranio-Maxillofacial Surgery, Klinikum Osnabrück GmbH, Am Finkenhügel 1, 49076 Osnabrück, Germany

**Keywords:** MTC, thyroid cancer, metastasis, medullary thyroid cancer, RET

## Abstract

**Background:** Medullary thyroid carcinoma (MTC) has a high rate of local and distant metastases. In particular, the RET protooncogene appears to be the predominant driver mutation for oncogenesis. The German S3 thyroid carcinoma guidelines recommend molecular genetic analysis of the tumour without specifying the site of the tissue sampling. Whether there is difference in RET protooncogene between the primary tumour, lymph node, and distant metastasis has not yet been investigated. However, differences could be important with regard to biopsy localization, and also, thus, the choice of single- or multi-tyrosine-kinase-inhibitor therapy. **Methods:** In a case of sporadic MTC, Cancer Hotspot panel diagnostics were performed on the primary tumour, lymph node metastasis, and distant metastasis. Mutations were classified using different gene databases, and the different stages of metastasis were compared. **Results:** RET protooncogene (*chr10:43609933, c.1886_1891delTGTGCG, p.Leu629_Asp631delinsHis*) was found to be present in the MTC tissue of the primary tumour, lymph node, and distant metastasis in the Cancer Hotspot Panel diagnostic, while the other investigated therapy-relevant mutational profiles were not consistently found. **Conclusions:** Further longitudinal studies in larger patient cohorts are required to elucidate the role of the RET protooncogene in the metastatic progression of MTC and to determine its impact on the selection of biopsy sites and the subsequent decision-making regarding single- versus multi-tyrosine kinase inhibitor therapy.

## 1. Introduction

Medullary thyroid carcinoma (MTC) is the third most common type of thyroid cancer after follicular and papillary thyroid carcinoma, and it is often neuroendocrinologically active [1]. MTC accounts for approx. 5–10% of all thyroid carcinomas, and the tumour genesis of MTC are parafollicular C-cells [2,3]. Ten-year survival is strongly dependent on the tumour stage (stage I: 100%; stage II: 93%; stage III: 71%; stage IV: 21%) [4]. MTC can be hereditary as an autosomal dominant MEN syndrome (multiple endocrine neoplasia syndromes) or occur randomly as a sporadic form [5]. Sporadic MTC occurs mainly between the ages of 40 and 60 [6].

Lymph node metastasis (LNM) and distant metastasis (DM) are common. LNM of the central compartment occur in about 50% of both sporadic and hereditary MTC [7]. The localization of DM can involve the lungs, bones, brain, or skin [8]. Mutations in cell signalling pathways, such as tyrosine kinases, also appear to play a role in cancer genesis. This clinically measurable tumour aggressiveness has a molecular biological basis that has been little-researched to date. Different mutations in RET, HRAS, and KRAS can be detected in MTC, and RET and RAS appear to be the predominant signalling pathways in MTC [9]. In addition to the interesting basic scientific and diagnostic data, therapies are also available. The multi-tyrosine inhibitors Cabozatinib and Vandetanib are possible therapy options approved by the EMA and FDA for advanced progressive and metastatic MTC [10,11]. Cabozatinib is a receptor tyrosine kinase inhibitor with activity against a broad range of targets, including MET, RET, AXL, VEGFR2, FLT3, and c-KIT [12]. The multi-tyrosine-kinase inhibitor Vandetanib targets RET, VEGFR, and EGFR [11]. Due to the sometimes considerable adverse effects of multi-tyrosine kinase inhibitors, the S3 guideline to be published in early 2025 recommends the preferential use of selective RET inhibitors in patients with advanced disease (symptomatic progression or radiologic evidence of disease progression), provided that a RET gene alteration is present. In phase I/II/III clinical trials, the selective RET inhibitor Selpercatinib demonstrated high response rates, with a significantly improved safety profile compared to Cabozatinib and Vandetanib. These favourable outcomes were observed both in treatment-naive patients and in those previously treated with tyrosine kinase inhibitors [13,14]. Other multi-tyrosine kinase inhibitors (Motesanib, Pazopanib, Sorafenib, Sunitinib, Lenvatinib) have also been described potential therapeutic relevant [15]. However, the options are currently not recommended. The homogeneity of the tumour cells is decisive or advantageous for systemic therapy with single-order multi-tyrosine-kinase inhibitors. However, it remains unclear whether molecular homogeneity exists between the primary tumour, LNM, and DM in advanced MTC. Comparative analyses at the mutational profile of the primary tumour, LNM and DM have not yet been published for advanced MTC. This information is crucial for the surgeon to choose the right location for the biopsy and for the treating oncologist to choose the right single- or multi-tyrosine-kinase-targeted therapy after a Cancer Hotspot Panel analysis of the MTC tissue. Whether this could influence the therapeutic consequences also remains to be elucidated.

This work is the first presentation of a Cancer Hotspot Panel analysis of a patient with sporadic MTC from the primary tumour to the LNM to the DM. The main question of this study is whether potential therapeutic consequences can be derived from the primary tumour or if the primary tumour shows tumour heterogeneity compared to LNM and/or DM.

## 2. Material and Methods

### 2.1. Institutional Review Board Statement

This study was approved by the local ethics committee (Ethical Committee of the Universitätsklinik RWTH Aachen Germany, approval no. EK23-341, 20 November 2023) and was conducted in accordance with the Guidelines for Good Clinical Practice and in compliance with the Declaration of Helsinki. An informed consensus was reached.

### 2.2. Tissue Samples and Patient

**Figure 1:**
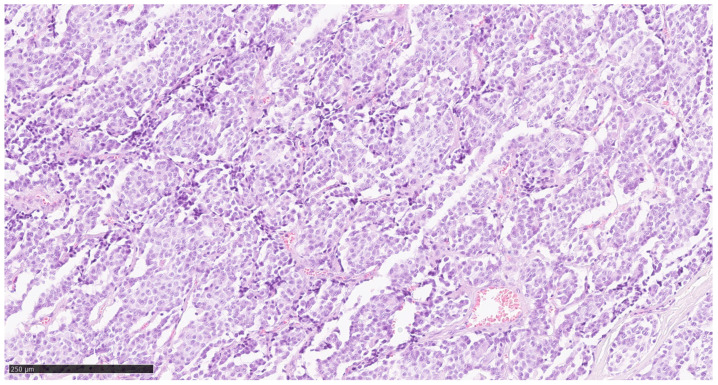
Shows a haematoxylin-eosin stained section of the MTC primary tumour tissue.

Tumour tissue was obtained from a histologically confirmed medullary thyroid carcinoma (MTC) at three anatomical sites: the primary tumour, a LNM, and a DM in the liver. The Cancer Hotspot Panel was applied to DNA material derived from formalin-fixed and paraffin-embedded (FFPE) tumour material (haematoxylin-eosin staining of primary tumour, 4 μm; Figure 1). The FFPE blocks containing the largest available tumour mass from the patient was selected for analysis. The initial diagnosis was made following the detection of a liver metastasis of unknown primary origin. Subsequent staging revealed a primary tumour in the thyroid gland accompanied by cervical LNM. A palliative thyroidectomy and cervical lymphadenectomy were performed to achieve local disease control, and histopathological confirmation of cervical LNM was obtained. Due to the advanced stage of disease and the patient’s poor general condition, systemic therapy could not be initiated. The patient was diagnosed with sporadic MTC. Genetic counselling was conducted, and multiple endocrine neoplasia (MEN) syndrome, as well as hereditary MTC, were excluded.

### 2.3. Next-Generation Sequencing: Cancer Hotspot Panel of MTC

DNA was isolated from the respective FFPE tissue block using the GeneRead DNA FFPE kit (Qiagen, Hilden, Germany) according to the manufacturer’s instructions. The quality of the DNA for Next Generation Sequencing (NGS) analysis was determined by functional testing of DNA amplifiability using a quantitative PCR specific for the RNaseP gene. A targeted mutation analysis was performed with the Ion Torrent PGM system (Life Technologies, Darmstadt, Germany) using the Cancer Hotspot Panel (Life Technologies). The following tumour genetic alterations were investigated in 50 oncogenes, for which targeted therapeutics are approved or known: ABL, AKT1, ALK, APC, ATM, BRAF, CDH1, CDKN2A, CSF1R, CTNNB1, EGFR, ERBB2, ERBB4, EZH2, FBW7, FGFR1, FGFR2, FGFR3, FLT3, GNA11, GNAS, GNAQ, HNF1A, HRAS, IDH1, IDH 2, JAK2, JAK3, KDR, KIT, KRAS, MET, MLH1, MPL, NOTCH1, NPM1, NRAS, PDGFRA, PIK3CA, PTEN, PTPN11, RB1, RET, SMAD4, SMARCB1, SMO, SRC, STK11, TP53, and VHL.

The analysis was performed using the Torrent Suite v. 5.0.5 software (Thermo fisher scientific, Darmstadt, Germany) and the ION REPORTER v.5.0 (Thermo fisher scientific). Excel 2016 for Windows by Microsoft (Redmond, WA, USA) was used for graphical representation.

### 2.4. Comparison with Gene Data Banks

All results and nomenclature are based on the human reference genome hg19. A search or comparison with the ClinVar (https://www.ncbi.nlm.nih.gov/clinvar/ (accessed on 14 July 2025)) and COSMIC (Catalogue of Somatic Mutations in Cancer) database was carried out. Additional nomenclatures of the multigene test, which form the basis of the evaluation, are presented below: coverage, dbSNP (reference SNP), SIFT (sorting intolerant from tolerant) [16], SNP (single nucleotide polymorphism), and PolyPhen (polymorphism phenotyping) [17].

## 3. Results

The Cancer Hotspot Panel analyses are presented in the direct assessment of whether the detected mutation is to be classified as benign or malignant. An overview of the raw data is shown in Table 1.

### 3.1. RET Protooncogene Is the Predominant Mutation in Cancer Hotspot Panel Analysis of Mtc Primary Tumour Tissue

For a comparative analysis of the sites of metastasis, the starting point of the metastasis (primaries; Figure 1) must first be analysed. The DNA analysis was performed with an average read depth (coverage) of 5804 reads/bp at coverage of 100% at 20× and 100% at 100×, which corresponds to excellent DNA quality. A mutation of the RET gene (chr10:43609933; c.1886_1891delTGTGCG; p.Leu629_Asp631delinsHis; coverage: 1983; allele frequency: 52.20%) was detected in the present sample. This mutation was not found exactly in the databases used, and is also described in the variant c.1884_1889delACTGTG. However, the mutation is located in a hotspot for pathogenic mutations. This area contains a large number of mutations that are classified as pathogenic in the databases. In addition, this mutation is classified as pathogenic in the literature [18]. It can, therefore, be assumed that this mutation has a pathogenic effect. The detected mutation of the RB1 gene (chr13:49033890; c.2039T>C, p.Ile680Thr; coverage: 1985; allele frequency: 5.33%) can be classified as pathogenic on the basis of the databases used. ClinVar has not recorded this mutation, but COSMIC assigns a pathogenic role to this mutation. In addition, alterations of the ERBB4, FGFR3, PDGFRA, KDR, APC, HMGXB3/CSF1R, EGFR, HRAS, FLT3, TP53, SMAD4, and STK11 genes, and a further alteration of the RET gene, were detected in this sample. The alteration c.215C>G, p.Pro72Arg of the TP53 gene leads to an amino acid exchange, which can be classified as non-pathogenic on the basis of the databases used. The variants of the FGFR3, PDGFRA, APC, EGFR, HRAS, and the other variant of the RET gene, do not lead to any changes in the amino acid sequence, and are to be classified as benign variants of the corresponding genes. The other alterations of the ERBB4, KDR, HMGXB3/CSF1R, EGFR, FLT3, SMAD, and STK11 genes are intronic variants whose relevance is unclear.

### 3.2. Mutational Profile Between the Primarius Tumour and the Lymph Node Metastases: RET Protooncogene Is the Predominant Mutation

To compare the gene alteration profile of the primarius tumour and LNM, we applied the same analysis to an MTC LNM of the same patient. The DNA analysis was also performed with an average read depth (coverage) of 4618 reads/bp with a coverage of 100% at 20× and 100% at 100×, which corresponds to excellent DNA quality. The same mutation of the RET gene (chr10:43609933, c.1886_1891delTGTGCG, p.Leu629_Asp631delinsHis, coverage: 1983, allele frequency: 48.32%) that was found in the primary tumour tissue was detected in the present sample. Furthermore, a mutation of the RET gene (chr10:43609936, c.1888T>C; p.Cys630Arg; coverage: 2000; allele frequency: 48.22%), was detected in the present sample, which is classified as pathogenic based on the databases used. Consistent with the primary tumour, the mutation of the RB1 gene (chr13:49033890; c.2039T>C; p.Ile680Thr; coverage: 1985; allele frequency: 6.30%), was detected and classified as pathogenic. In addition, pathogenic mutations were detected, but these only have a very low allele frequency of 1–2% and their relevance must, therefore, be critically assessed in the following genes: EGFR (chr7:55221821; c.865G>A; p.Ala289Thr; coverage: 2000; allele frequency: 1.28%) and FLT3 (chr13:28592620; c.2525A>G; p.Tyr842Cys; coverage: 2000; allele frequency: 1.50%).

Moreover, alterations of the ERBB4, FGFR3, PDGFRA, KDR, APC, HMGXB3/CSF1R, EGFR, MET, HRAS, FLT3, TP53, SMAD4, STK11, GNAS, and a further alteration of the RET gene were detected in the present sample. The detected variants c.535G>A, p.Ala179Thr and c.1099A>G, p.Ile367Val of the MET gene lead to changes in the amino acid sequence that can be classified as non-pathogenic on the basis of the databases used. The alteration c.215C>G, p.Pro72Arg of the TP53 gene leads to an amino acid exchange, which can be classified as non-pathogenic. Furthermore, the alteration c.2525A>G, p.Tyr842Cys of the FLT3 gene can also be classified as a non-pathogenic variant. The variants of the FGFR3, PDGFRA, APC, EGFR, HRAS, and the other variant of the RET gene do not lead to any changes in the amino acid sequence, and are, hence, to be classified as benign variants of the corresponding genes. The alterations of the ERBB4, KDR, HMGXB3/CSF1R, EGFR, FLT3, SMAD4, STK11, and GNAS genes are intronic variants whose relevance is unclear.

**Figure 2:**
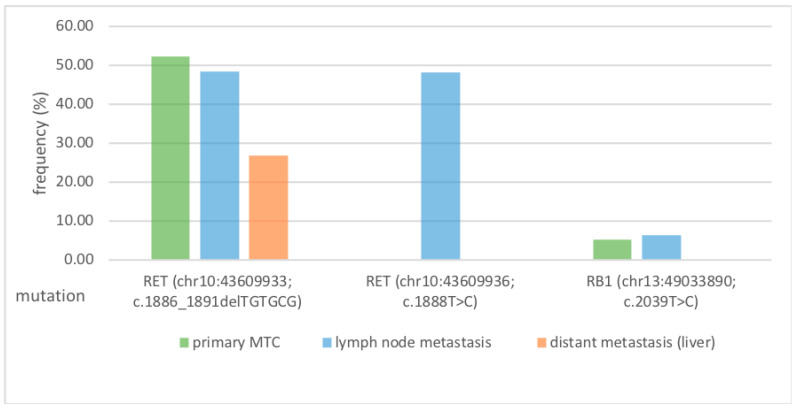
RET-protooncogene (chr10:43609933, c.1886_1891delTGTGCG) is the dominant mutation in primary tumour tissue, lymph-node metastasis, and distant metastasis, and is potentially involved in metastasis genesis. The figure Shows the pathogenic-relevant mutational profile (mutation (locus; coding) of Cancer Hotspot Panel analysis of primary MTC, lymph node metastasis, and distant metastasis (specified in frequency%). The RET mutation (chr10:43609933; c.1886_1891delTGTGCG) was found in all stations of metastasis (left side). In addition, the RET (chr10:43609936; c.1888T>C) mutation was detected only in the isolated lymph node metastasis (centre). This isolated finding appears to play a subordinate role due to the heterogeneity compared to primary MTC and distant metastasis. The RB1 (chr13:49033890; c.2039T>C) mutation, which is only found in primary MTC and lymph node metastasis, also appears to play a subordinate role (right side). In summary, the RET mutation (chr10:43609933; c.1886_1891delTGTGCG) may be of particular interest for future investigations into the metastatic progression of medullary thyroid carcinoma, as it was detected across all stages of metastasis, suggesting tumour homogeneity in this case.

In summary of the analysis of the primary tumour vs. LNM, the evaluation of the mutation classified as reliably pathogenic (RET gene (chr10:43609933, c.1886_1891delTGTGCG; RB1 gene, chr13:49033890, c.2039T>C) shows a match between primary tumour tissue and LNM. Nevertheless, the RET gene mutation (chr10:43609933, c.1886_1891delTGTGCG) was clearly more frequently detectable and can, therefore, be proven to be homogenous (Table 1, Figure 2).

### 3.3. RET Protooncogene Is Independent of the Metastasis Localization

Once it had been established that primarius and LNM differed only minimally in their mutational profile, the comparative analysis for MTC DM still had to be carried out. The DNA analysis was performed with an average read depth (coverage) of 6524 reads/bp with a coverage of 100% at 20× and 100% at 100×, which corresponds to excellent DNA quality. The same mutation as in the primary tumour and LNM (RET gene (chr10:43609933; c.1886_1891delTGTGCG; p.Leu629_Asp631delinsHis; coverage: 1983; allele frequency: 26.83%)) was detected in the present sample of DM. In addition, alterations of the ERBB4, FGFR3, PDGFRA, KDR, APC, EGFR, NOTCH1, HRAS, FLT3, TP53, SMAD4, and STK11, and a further alteration of the RET gene, were detected in the present sample. The alterations of the FGFR3, PDGFRA, APC, EGFR, and HRAS, and a further alteration of the RET gene, do not lead to any change in the amino acid sequence, and are to be classified as benign variants of the corresponding genes. The alterations of the ERBB4, KDR, FLT3, SMAD4, and STK11 genes are intronic variants whose relevance is unclear. The alteration of the TP53 gene leads to an amino acid exchange that can be classified as non-pathogenic based on the databases used. The alteration of the NOTCH1 gene leads to an amino acid exchange, the effect of which is not recorded in the databases used.

When evaluating the pathogenic mutations of DM, a match can also be found in the RET protooncogene compared to the primary tumour tissue and the LNM. Interestingly, the RB1 mutation disappears, and the second variant of the RET protooncogene can also no longer be detected.

### 3.4. Summary of Results

RET was found to be the dominant mutation of the primary tumour from LNM to DM. It appears that, in these back-to-back analyses from the same patient, there is less heterogeneity between primary MTC tissue and metastases. Figure 2 shows a comparative overview of the tumour-specific and therapy-relevant pathogenic mutations.

## 4. Discussion

### 4.1. Evaluation of the Pathogenicity of the Mutations

Various mutations for the RET protooncogene have been described in the literature [19,20,21,22]. When assessing which of the mutations contribute to metastasis progression, a homogeneous occurrence of the mutation in the primary MTC and the localization of metastasis is a prerequisite. Therefore, the pathogenic RET (chr10:43609936; c.1888T>C) and the pathogenic RB1 (chr13:49033890; c.2039T>C) mutation were considered incidental findings and are perhaps less involved in metastasis genesis or metastasis progression. The INDEL mutation (p.Leu629_Asp631delinsHis) occurring in this case is also described in the literature [23]. Due to the homogeneity in the metastasis (primary MTC, LNM, DM), the occurrence of this RET mutation appears to be a possible cause of the involvement of metastasis genesis or metastasis progression. Given that this observation is based on a single patient (n = 1), the findings remain speculative, and the hypothesis should be further investigated in longitudinal studies.

Notably, the p.C630R mutation is commonly associated with familial MTC and MEN2A syndrome [24]. There is currently no explanation as to why this mutation is exclusively detected in the LNM and is present in this case of sporadic MTC. However, its clinical significance appears to be limited, as the mutation was not identified in the other metastasis sites. Nevertheless, it is interesting to note that the gene RB1, which has been described individually as pathogenic, differ in the various locations. RB1 is present in the primary tumour and LNM and then disappears in DM. The loss of the mutated gene can be visualised here. The loss of the mutated gene is to be assumed here, as the frequency of the RET protooncogene also decreases from the primary tumour via LNM and DM.

### 4.2. RET Protooncogene Is the Predominant Mutation from Primary Tumour to Lymph Node Metastasis to Distant Metastasis—What Therapeutic Relevance Does This Have?

The primary objective of this study was to determine whether differences in RET protooncogene and other potential therapeutic targets depend on the biopsy site, and whether this may have therapeutic implications. On the one hand, it may be argued that the biopsy site is of limited relevance for surgeons, pathologists, and oncologists when determining subsequent treatment with selective or multi-tyrosine-kinase inhibitors. In this patient, the homogeneous detection of RET across different tumour sites supports this view. On the other hand, one could argue that the biopsy approach should be as accessible as possible, favouring the primary tumour as the preferred site. Furthermore, differential clinical local progression of individual metastases may occur, potentially justifying the selection of specific metastatic sites for biopsy. Ultimately, this remains an individualised, case-by-case clinical decision. The RET protooncogene appears to be the most important driver mutation, as it occurs in all three localizations. However, follow-up studies must verify this hypothesis with a broader spectrum of tumour samples. In order to create the basis for such studies, the collection of tumour samples from the metastases of rare tumour entities, such as MTC, in centralised biobanks, as well as cooperation with various translational oncology disciplines, is of crucial importance. According to the current German S3 guidelines for thyroid carcinomas (2025), a molecular genetic analysis should be performed in cases of sporadic medullary thyroid carcinoma (MTC) prior to the initiation of systemic therapy. This is to assess the possibility of administering a selective RET inhibitor therapy or a treatment with Vandetanib. The guidelines do not address the localization of the biopsy site, which underscores the relevance of the present study. Due to its low side-effect profile, the selective RET inhibitor Selpercatinib should be used [13,14]. The low intratumoural heterogeneity within the tumour cell population supports the use of targeted RET inhibition in advanced-stage patients, as exemplified by the case presented.

### 4.3. Strengths and Weaknesses of This Study

A weakness of this study is that only one patient was included, and the observed associations need to be analysed in larger collectives. The conclusion of the study presented here is very limited and must be critically evaluated. Nevertheless, this study is valuable for subsequent studies due to the rare simultaneous presence of the samples (primary tumour, LNM, and DM) in the context of a low incidence of MTC. There appears to be limited tumour heterogeneity between the primary tumour site, lymph node metastases (LNM), and distant metastases (DM). If confirmed by further studies, this may indicate a high degree of homogeneity with respect to the RET protooncogene within the tumour. This observation has potential diagnostic and therapeutic implications both for surgeons and oncologists, underscoring the significance of these findings. In addition to the selection of the site for mutation analysis in medullary thyroid carcinoma (MTC), genetic syndromes—such as multiple endocrine neoplasia (MEN) syndromes and hereditary MTC—may also influence the molecular results. The sample analysed in this study was derived from a patient with sporadic MTC. Nevertheless, it remains an open question whether RET mutations exhibit similar consistency or variability across different metastatic sites in patients with MEN syndromes.

## 5. Conclusions

This study is the first to show, in one MTC patient, that the RET mutation (chr10:43609933, c.1886_1891delTGTGCG, p.Leu629_Asp631delinsHis) can be identified from the primary MTC via the LNM to the DM. It appears that the RET protooncogene may play the decisive role in the development of metastases or metastasis progression in MTC, a hypothesis that needs to be investigated in larger cohorts. Based on our data in one patient, the choice of biopsy site in advanced MTC does not seem to be decisive for the choice of targeted therapy. Nevertheless, further studies are needed to verify this hypothesis in larger cohorts.

### Highlights

First analysis and comparison of the therapy-relevant mutation profile, including a Cancer Hotspot Panel, of a medullary thyroid carcinoma in the localizations of metastasis: primary, lymph node, and distant metastasis. The RET gene (chr10:43609933, c.1886_1891delTGTGCG, p.Leu629_Asp631delinsHis) was detected in all three stages of metastasis. Owing to the observed homogeneity of the tumour tissue, the biopsy site appears to be of limited relevance and does not adversely affect the selection of multi-tyrosine-kinase inhibitor therapy. However, as this finding is derived from a single-case analysis, it remains a hypothesis that warrants validation in larger, prospective studies.

## Figures and Tables

**Table 1:** Overview of raw data of relevant Cancer Hotspot analysis. (n.d. = not detectable).

							Distant Metastasis MTC	Lymph Node Metastasis MTC	Primary Tumour MTC					
**Locus**	**Potential Targets**	Coding	Exon	Transcript	Amino Acid Change	Coverage	% Frequency	% Frequency	% Frequency	Variant ID	ClinVar	Type	Length	Variant Effect
chr2:212812097	ERBB4	c.421+58A>G		NM_005235.2	p.?	1999	99.90	100.00	100.00			SNV	1	unknown
chr4:1807894	FGFR3	c.1953G>A	14	NM_000142.4	p.(=)	1999	99.75	99.90	99.85			SNV	1	synonymous
chr4:55141050	PDGFRA	c.1701A>G	12	NM_006206.4	p.(=)	1984	99.90	100.00	99.95	COSM12417		SNV	1	synonymous
chr4:55980239	KDR	c.798+54G>A		NM_002253.2	p.?	1329	99.70	100.00	99.90			SNV	1	unknown
chr5:112175769	APC	c.4479G>A	16	NM_000038.5	p.(=)	1989	45.55	48.48	53.19	COSM19714; COSM19626; COSM19349; COSM19674; COSM23598		SNV	1	synonymous
chr5:149433596	CSF1R, HMGXB3	c.*1841TG>GA, c.2954_2955delCAinsTC		NM_014983.2, NM_005211.3	p.?, p.?	1847	99.78	99.93	100.00			MNV	2	unknown, unknown
chr7:55249063	EGFR, EGFR-AS1	c.2361G>A	20	NM_005228.3, NR_047551.1	p.(=)	1592	49.25	53.82	53.41		Benign, Likely benign	SNV	1	synonymous
chr10:43609933	RET	c.1886_1891delTGTGCG	11	NM_020975.4	p.Leu629_Asp631delinsHis	1983	26.83	48.32	52.20	COSM27040		INDEL	6	non- frameshift deletion
chr10:43609936	RET	c.1888T>C	11	NM_020975.4	p.Cys630Arg	1999	n.d.	48.22	n.d.	1237917:964:29806	Pathogenic: Likely pathogenic	SNV	1	missense
chr10:43613843	RET	c.2307G>T	13	NM_020975.4	p.(=)	1998	100.00	100.00	99.90		Benign	SNV	1	synonymous
chr11:534242	HRAS	c.81T>C	2	NM_001130442.2	p.(=)	2000	45.70	52.65	55.53	COSM249860	Benign	SNV	1	synonymous
chr13:28610183	FLT3	c.1310-3T>C		NM_004119.2	p.?	2000	68.75	57.05	50.10			SNV	1	unknown
chr13:49033890	RB1	c.2039T>C	20	NM_000321.2	p.Ile680Thr	1985	n.d.	6.30	5.33	870		SNV	1	missense
chr17:7579472	TP53	c.215C>G	4	NM_000546.5	p.Pro72Arg	2000	99.25	99.97	99.22	COSM45985	Benign. Uncertain significance, drug response	SNV	1	missense
chr18:48586344	SMAD4	c.955+58C>T		NM_005359.5	p.?	1997	50.13	48.47	50.70			SNV	1	unknown
chr19:1220321	STK11	c.465-51T>C		NM_000455.4	p.?	1707	47.51	50.39	47.72			SNV	1	unknown

## Data Availability

The data or parts of the data presented in this study are available on request from the corresponding author. There is a restriction due to data protection, meaning that the overall data is not publicly accessible.

## Abbreviations

ClinVar	ClinVar Databank
COSMIC	catalogue of somatic mutations in cancer; coverage
dbSNP	reference Single nucleotide polymorphism
DM	distant metastasis
EMA	European Medicines Agency
FDA	U.S. Food and Drug Administration
LNM	lymph node metastasis
MTC	medullary thyroid cancer
MEN	multiple endocrine neoplasia
NRTK	neurotropic tyrosine receptor kinase
RB1	Tumour suppressor Retinoblastom Protein 1
RET	Receptor tyrosine kinase RET
SIFT	sorting intolerant from tolerant
SNP	single nucleotide polymorphism
PolyPhen	polymorphism phenotyping
